# Adjusting Sample Sizes for Different Categories of Embodied Cognition Research

**DOI:** 10.3389/fpsyg.2018.02384

**Published:** 2018-12-20

**Authors:** Alexander Skulmowski, Günter Daniel Rey

**Affiliations:** Psychology of Learning with Digital Media, Chemnitz University of Technology, Chemnitz, Germany

**Keywords:** cognitive psychology, embodied cognition, replicability, methodology, statistics

## Introduction

Research in the field of embodied cognition is occupied with a variety of research questions stemming from the idea that cognition is deeply connected with bodily aspects such as perception and action (Barsalou, 1999, 2008). However, some embodiment studies have been identified to exhibit problems such as non-replicable results (Lakens, 2014). With this article, we wish to accomplish three aims: exemplifying ways of categorizing embodied cognition research in an informative manner; providing guidelines on how to identify problematic study designs; suggesting solutions for potentially problematic designs.

Within the field of embodied cognition, several aspects are investigated as outlined by Wilson (2002). One example for embodiment mentioned by Wilson (2002) is gesturing (for an overview on gesturing, see Hostetter and Alibali, 2008). Embodied cognition theory can be used to analyze the relation between gestures and mental processes (e.g., Hostetter and Alibali, 2008). Furthermore, there is a debate around the question whether language and meaning are *grounded* in perceptual contents experienced through the body (e.g., Borghi et al., 2004; for an overview on grounded cognition, see Barsalou, 2010). Besides research on cognition, principles of embodied cognition have been applied to fields such as social psychology (see Meier et al., 2012, for an overview) and educational psychology (see Paas and Sweller, 2012, for an overview). For instance, research on embodiment in the context of social cognition has provided evidence for the claim that bodily sensations such as weight can alter judgments on importance (e.g., Ackerman et al., 2010). In educational psychology, one application of embodiment theory is the design of interactive learning environments (e.g., Johnson-Glenberg et al., 2014).

In response to the current *replication crisis* in psychology (for discussions, see Pashler and Wagenmakers, 2012; Maxwell et al., 2015), several solutions have been proposed to improve the quality of psychological research (e.g., Chambers, 2013; Simons, 2014; LeBel, 2015; for overviews, see Ferguson, 2015; Zwaan et al., 2017). Benjamin et al. (2018) argue for a change of the standard 0.05 alpha level and instead support to lower the default alpha value for novel findings in the field of psychology to 0.005. Importantly, the sample size and power of studies have been described as pivotal contributors to replicable results (Fraley and Vazire, 2014).

Multiple types of embodied cognition research are facing the problem of delivering non-replicable results as discussed in the literature (e.g., Rabelo et al., 2015). Perugini et al. (2014) present a method for the calculation of sample sizes for replication studies and confirmatory research that takes into account that observed effect sizes may be inaccurate estimates. They suggest to conduct sample size calculations using an effect size that is based on the lower bounds of the confidence interval computed for an observed effect size (Perugini et al., 2014). Another method is presented by Simonsohn (2015), who makes the argument that sample size calculations for replication studies should not merely use the effect sizes reported in the original research that is to be replicated. He explains that by increasing the sample size by the factor of 2.5, a replication study can be used to assess whether an effect is too small to have been appropriately captured in the original study (Simonsohn, 2015). This method has already been used in a recent replication study on embodied cognition effects (Ronay et al., 2017). We suggest to use one of the aforementioned methods of sample size calculation for studies involving embodiment-based manipulation types that are known for potential problems. In the following, we will present three important aspects that can be used to check whether an embodied cognition study design will need amendments such as an increased sample size.

## Identifying Problematic Embodiment Research

We wish to present three ways of assessing embodied cognition research: (1) categorization in reference to the dimensions of *bodily engagement* and *task integration* (based on Skulmowski and Rey, 2018); (2) categorization using the *directness* of an embodiment manipulation (based on ideas presented by Lee and Schwarz, 2014); (3) considering moderators and boundary conditions (based on Fay and Maner, 2015; see also, e.g., Maglio and Trope, 2012; Kaspar et al., 2016; Skulmowski and Rey, 2017a).

### Bodily Engagement and Task Integration

Skulmowski and Rey (2018) presented a taxonomy of *embodied learning* research that hinges upon the two dimensions bodily engagement and task integration. They define low levels of bodily engagement as tasks with only minor bodily movement that occurs while sitting (Skulmowski and Rey, 2018). High levels of bodily engagement are considered to be tasks in which locomotion or other extensive forms of bodily movement are required (Skulmowski and Rey, 2018). Skulmowski and Rey (2018) ground the notion of task integration in Wilson and Golonka's (2013) characterization of embodied cognition as a task-bound utilization of bodily resources. In the taxonomy of Skulmowski and Rey (2018), *incidental* manipulations are said to be reliant on cues that alter cognitive variables while Skulmowski and Rey (2018) state *integrated* manipulations to be strongly intertwined with tasks.

Although the taxonomy of Skulmowski and Rey (2018) is mainly focused on embodied learning, in this paper we highlight applications of this taxonomy to decisions concerning virtually all types of embodied cognition research. Most importantly, Skulmowski and Rey (2018) discuss how the power of a study can be affected by the location of the study on the matrix of their taxonomy. By comparing the results of two study series utilizing similar types of incidental weight manipulations in a learning task (Alban and Kelley, 2013; Skulmowski and Rey, 2017a), Skulmowski and Rey (2018) conclude that a higher degree of bodily engagement can increase effect sizes. Turning to the factor of task integration, based on studies such as Mavilidi et al. (2015), Skulmowski and Rey (2018) state that integrated embodied learning manipulations have led to better learning results compared to non-integrated forms in which an embodiment manipulation does not have a connection to the learning contents. Based on these two conclusions regarding the two dimensions of the taxonomy, we derive the assumption that embodied cognition research may in general thought to be most robust when both the level of bodily engagement and the degree of task integration are high. Conversely, as stated by Skulmowski and Rey (2018), incidental manipulations with minor bodily engagement can lead to smaller effects. In addition, such designs may even result in non-reproducible effects (e.g., Rabelo et al., 2015). However, we emphasize that meta-analyses are necessary to provide concrete evidence concerning the relation between the robustness of results and the location on the grid of the 2 × 2 taxonomy (i.e., low bodily engagement + high task integration and high bodily engagement + low task integration). In sum, study designs which are low both in bodily engagement and task integration according to the taxonomy of Skulmowski and Rey (2018) should be considered as potentially problematic.

### Categorization Based on Directness

Besides embodiment studies that are focused on tasks, others revolve around metaphor-based effects and priming (Wilson and Golonka, 2013). An appropriate way to categorize this type of study relies on the directness of the embodiment manipulation as defined by Lee and Schwarz (2014). Lee and Schwarz (2014) discuss embodied cognition studies based on Lakoff and Johnson's (1999) metaphor model of cognition. Lakoff and Johnson's (1999) theory states that conceptual knowledge heavily relies on metaphors and that cognitive processes are influenced by the body. Lee and Schwarz (2014) summarize several metaphoric relations that have been utilized in embodied cognition research, such as the idea that “fishy” smells are connected to the feeling of suspicion based on the corresponding metaphorical relation found in the English language (they cite Lee and Schwarz, 2012, as an example). In addition, Lee and Schwarz (2014) present several types of embodiment studies (see Skulmowski and Rey, 2017a, for a discussion of these types). Lee and Schwarz (2014) acknowledge that there are embodiment effects not relying on metaphors, such as physiological effects, and call them “[d]irect, non-metaphoric effects” (p. 100, italics removed). Furthermore, among other types, they describe metaphor-based studies which affect judgments based on how the perception of a setting is manipulated by bodily, metaphor-based influences (Lee and Schwarz, 2014). In addition, Lee and Schwarz (2014) point out that there are metaphor-based embodiment effects that depend on participants' lack of awareness of the embodiment manipulation. It needs to be noted that Lee and Schwarz (2014) discuss these aspects as they relate to incidental study designs with a focus on the field of decision-making. However, we want to generalize certain aspects of their overview for the entire field of embodied cognition research. Embodied cognition research could be viewed on a continuum ranging from direct effects as defined by Lee and Schwarz (2014) to indirect effects (comparable to the metaphorical effects described by Lee and Schwarz, 2014).

Lakens (2014) reviews several failed replication attempts that match the criteria for indirect effects and concludes that one may have doubts concerning such types of embodiment studies. In line with Laken's (2014) statements, we support the idea that studies falling on the indirect end of the spectrum should use larger sample sizes. Generally speaking, there have been several criticisms directed toward study designs involving metaphor-based manipulations (e.g., Lakens, 2014; Pecher, 2017; Skulmowski and Rey, 2017a). Based on these grounds, we argue to treat such designs as potentially problematic.

### Adjustments Based on Moderating Factors and Boundary Conditions

Fay and Maner (2015) recently used moderating factors and boundary conditions as an explanation for the non-replicability of certain embodiment effects. According to their view, instances in which findings concerning embodiment manipulations were not replicable may be the result of moderators or boundary conditions that were not considered (Fay and Maner, 2015). Therefore, they argue for giving more thought to moderators and boundary conditions in embodiment research (Fay and Maner, 2015). Recent examples include the moderating factor gender (e.g., Kaspar et al., 2016) as well as the effects of the cognitive mode activated during a task (Maglio and Trope, 2012; Skulmowski and Rey, 2017a).

In addition, it should be noted that some types of measures are more appropriate for embodied cognition research than others (Meier et al., 2012), for example when assessing cognitive load (Skulmowski and Rey, 2017b). In sum, researchers should be aware of potential moderators and boundary conditions when planning their embodiment studies.

## Conclusion

In light of the current controversy surrounding the replicability of psychological science, we wish to emphasize a variety of aspects that may warrant a higher sample size or other amendments to embodied cognition study designs. One method of assessment concerns the degree of bodily engagement and the extent to which an embodiment manipulation affects how a task can be solved as described by Skulmowski and Rey (2018). Another method of categorizing studies presented in this paper is a continuum between direct and indirect effects (based on theoretical considerations of Lee and Schwarz, 2014). As outlined above, strengthening the factor of bodily engagement may increase effect sizes (Skulmowski and Rey, 2018). Lastly, we emphasized that embodied cognition study designs should check for moderating effects and boundary conditions as studies (e.g., Skulmowski and Rey, 2017a) have demonstrated that even strong bodily manipulations need to be directed at suitable processing modes. These three factors clearly are not an exhaustive list of factors that need to be kept in mind when planning an embodied cognition study. However, we argue that the three aspects discussed in this paper may help to identify potentially problematic study designs; and that using one of the methods described by Perugini et al. (2014) and Simonsohn (2015) could help to improve embodiment research. Additional research needs to be done to determine more precise guidelines based on meta-analyses that could be grounded in the models presented in this paper.

## Author Contributions

AS and GDR drafted and revised the article and approved the submitted version for publication.

### Conflict of Interest Statement

The authors declare that the research was conducted in the absence of any commercial or financial relationships that could be construed as a potential conflict of interest.

## References

[B1] Ackerman J. M. Nocera C. C. Bargh J. A. (2010). Incidental haptic sensations influence social judgments and decisions. Science 328, 1712–1715. 10.1126/science.1189993 20576894 PMC3005631

[B2] Alban M. W. Kelley C. M. (2013). Embodiment meets metamemory: weight as a cue for metacognitive judgments. J. Exp. Psychol. Learn. Mem. Cogn. 39, 1628–1634. 10.1037/a0032420 23565793

[B3] Barsalou L. W. (1999). Perceptions of perceptual symbols. Behav. Brain Sci. 22, 637–660. 10.1017/S0140525X99532147 11301525

[B4] Barsalou L. W. (2008). Grounded cognition. Annu. Rev. Psychol. 59, 617–645. 10.1146/annurev.psych.59.103006.093639 17705682

[B5] Barsalou L. W. (2010). Grounded cognition: past, present, and future. Top. Cogn. Sci. 2, 716–724. 10.1111/j.1756-8765.2010.01115.x 25164052

[B6] Benjamin D. J. Berger J. O. Johannesson M. Nosek B. A. Wagenmakers E. J. Berk R. (2018). Redefine statistical significance. Nat. Hum. Behav. 2, 6–10. 10.1038/s41562-017-0189-z 30980045

[B7] Borghi A. M. Glenberg A. M. Kaschak M. P. (2004). Putting words in perspective. Mem. Cogn. 32, 863–873. 10.3758/BF03196865 15673175

[B8] Chambers C. D. (2013). Registered reports: a new publishing initiative at Cortex. Cortex 49, 609–610. 10.1016/j.cortex.2012.12.016 23347556

[B9] Fay A. J. Maner J. K. (2015). Embodied effects are moderated by situational cues: warmth, threat, and the desire for affiliation. Brit. J. Soc. Psychol. 54, 291–305. 10.1111/bjso.12088 25302629

[B10] Ferguson C. J. (2015). “Everybody knows psychology is not a real science”: Public perceptions of psychology and how we can improve our relationship with policymakers, the scientific community, and the general public. Am. Psychol. 70, 527–542. 10.1037/a0039405 26348335

[B11] Fraley R. C. Vazire S. (2014). The N-pact factor: evaluating the quality of empirical journals with respect to sample size and statistical power. PLoS ONE 9:e109019. 10.1371/journal.pone.0109019 25296159 PMC4189949

[B12] Hostetter A. B. Alibali M. W. (2008). Visible embodiment: gestures as simulated action. Psychon. Bull. Rev. 15, 495–514. 10.3758/PBR.15.3.495 18567247

[B13] Johnson-Glenberg M. C. Birchfield D. A. Tolentino L. Koziupa T. (2014). Collaborative embodied learning in mixed reality motion-capture environments: two science studies. J. Educ. Psychol. 106, 86–104. 10.1037/a0034008

[B14] Kaspar K. Jurisch A. Schneider M. (2016). Embodied cognition and humor: the impact of weight sensations on humor experience and the moderating role of gender. Curr. Psychol. 35, 377–385. 10.1007/s12144-015-9304-3

[B15] Lakens D. (2014). Grounding social embodiment. Soc. Cogn. 32, 168–183. 10.1521/soco.2014.32.supp.168

[B16] Lakoff G. Johnson M. (1999). Philosophy in the Flesh: The Embodied Mind and Its Challenge to Western Thought. New York, NY: Basic Books.

[B17] LeBel E. P. (2015). A new replication norm for psychology. Collabra. 1:4 10.1525/collabra.23

[B18] Lee S. W. S. Schwarz N. (2012). Bidirectionality, mediation, and moderation of metaphorical effects: the embodiment of social suspicion and fishy smells. J. Pers. Soc. Psychol. 103, 737–749. 10.1037/a0029708 22905770

[B19] Lee S. W. S. Schwarz N. (2014). “Metaphors in judgment and decision making,” in The Power of Metaphor: Examining Its Influence on Social Life, eds Landau M. J. Robinson M. D. Meier B. P. (Washington, DC: APA), 85–108.

[B20] Maglio S. J. Trope Y. (2012). Disembodiment: abstract construal attenuates the influence of contextual bodily state in judgment. J. Exp. Psychol. Gen. 141, 211–216. 10.1037/a0024520 21767044 PMC3199306

[B21] Mavilidi M. F. Okely A. D. Chandler P. Cliff D. P. Paas F. (2015). Effects of integrated physical exercises and gestures on preschool children's foreign language vocabulary learning. Educ. Psychol. Rev. 27, 413–426. 10.1007/s10648-015-9337-z

[B22] Maxwell S. E. Lau M. Y. Howard G. S. (2015). Is psychology suffering from a replication crisis? What does “failure to replicate” really mean? Am. Psychol. 70, 487–498. 10.1037/a0039400 26348332

[B23] Meier B. P. Schnall S. Schwarz N. Bargh J. A. (2012). Embodiment in social psychology. Top. Cogn. Sci. 4, 705–716. 10.1111/j.1756-8765.2012.01212.x 22777820

[B24] Paas F. Sweller J. (2012). An evolutionary upgrade of cognitive load theory: using the human motor system and collaboration to support the learning of complex cognitive tasks. Educ. Psychol. Rev. 24, 27–45. 10.1007/s10648-011-9179-2

[B25] Pashler H. Wagenmakers E. J. (2012). Editors' introduction to the special section on replicability in psychological science: a crisis of confidence? Perspect. Psychol. Sci. 7, 528–530. 10.1177/1745691612465253 26168108

[B26] Pecher D. (2017). Curb Your Embodiment. Top. Cogn. Sci. 10, 501–517. 10.1111/tops.12311 29214726

[B27] Perugini M. Gallucci M. Costantini G. (2014). Safeguard power as a protection against imprecise power estimates. Perspect. Psychol. Sci. 9, 319–332. 10.1177/1745691614528519 26173267

[B28] Rabelo A. L. Keller V. N. Pilati R. Wicherts J. M. (2015). No effect of weight on judgments of importance in the moral domain and evidence of publication bias from a meta-analysis. PLoS ONE 10:e0134808. 10.1371/journal.pone.0134808 26241042 PMC4524628

[B29] Ronay R. Tybur J. M. van Huijstee D. Morssinkhof M. (2017). Embodied power, testosterone, and overconfidence as a causal pathway to risk-taking. Comprehens. Results Soc. Psychol. 2, 28–43. 10.1080/23743603.2016.1248081

[B30] Simons D. J. (2014). The value of direct replication. Perspect. Psychol. Sci. 9, 76–80. 10.1177/1745691613514755 26173243

[B31] Simonsohn U. (2015). Small telescopes: detectability and the evaluation of replication results. Psychol. Sci. 26, 559–569. 10.1177/0956797614567341 25800521

[B32] Skulmowski A. Rey G. D. (2017a). Bodily effort enhances learning and metacognition: investigating the relation between physical effort and cognition using dual-process models of embodiment. Adv. Cogn. Psychol. 13, 3–10. 10.5709/acp-0202-9 28439318 PMC5383935

[B33] Skulmowski A. Rey G. D. (2017b). Measuring cognitive load in embodied learning settings. Front. Psychol. 8:1191. 10.3389/fpsyg.2017.01191 28824473 PMC5539229

[B34] Skulmowski A. Rey G. D. (2018). Embodied learning: introducing a taxonomy based on bodily engagement and task integration. Cogn. Res. Principles Implicat. 3:6. 10.1186/s41235-018-0092-9 29541685 PMC5840215

[B35] Wilson A. D. Golonka S. (2013). Embodied cognition is not what you think it is. Front. Psychol. 4:58. 10.3389/fpsyg.2013.00058 23408669 PMC3569617

[B36] Wilson M. (2002). Six views of embodied cognition. Psychon. Bull. Rev. 9, 625–636. 10.3758/BF03196322 12613670

[B37] Zwaan R. A. Etz A. Lucas R. E. Donnellan M. B. (2017). Making replication mainstream. Behav. Brain Sci. 41:e120 10.1017/S0140525X17001972 29065933

